# Role of Vitamin D in Cognitive Dysfunction: New Molecular Concepts and Discrepancies between Animal and Human Findings

**DOI:** 10.3390/nu13113672

**Published:** 2021-10-20

**Authors:** Zsolt Gáll, Orsolya Székely

**Affiliations:** 1 Department of Pharmacology and Clinical Pharmacy, George Emil Palade University of Medicine, Pharmacy, Science and Technology of Târgu Mureș, 540142 Târgu Mureș, Romania; 2 Department of Nephrology/Internal Medicine, Mures County Clinical Hospital, 540103 Târgu Mureș, Romania; gall.orsolya525@gmail.com

**Keywords:** vitamin D, vitamin D deficiency, central nervous system, cognitive function, brain development

## Abstract

Purpose of review: increasing evidence suggests that besides the several metabolic, endocrine, and immune functions of 1alpha,25-dihydroxyvitamin D (1,25(OH)2D), the neuronal effects of 1,25(OH)2D should also be considered an essential contributor to the development of cognition in the early years and its maintenance in aging. The developmental disabilities induced by vitamin D deficiency (VDD) include neurological disorders (e.g., attention deficit hyperactivity disorder, autism spectrum disorder, schizophrenia) characterized by cognitive dysfunction. On the other hand, VDD has frequently been associated with dementia of aging and neurodegenerative diseases (e.g., Alzheimer’s, Parkinson’s disease). Recent findings: various cells (i.e., neurons, astrocytes, and microglia) within the central nervous system (CNS) express vitamin D receptors (VDR). Moreover, some of them are capable of synthesizing and catabolizing 1,25(OH)2D via 25-hydroxyvitamin D 1alpha-hydroxylase (CYP27B1) and 25-hydroxyvitamin D 24-hydroxylase (CYP24A1) enzymes, respectively. Both 1,25(OH)2D and 25-hydroxyvitamin D were determined from different areas of the brain and their uneven distribution suggests that vitamin D signaling might have a paracrine or autocrine nature in the CNS. Although both cholecalciferol and 25-hydroxyvitamin D pass the blood–brain barrier, the influence of supplementation has not yet demonstrated to have a direct impact on neuronal functions. So, this review summarizes the existing evidence for the action of vitamin D on cognitive function in animal models and humans and discusses the possible pitfalls of therapeutic clinical translation.

## 1. Introduction

Vitamin D deficiency (VDD) affects almost one billion people today [1]. It is now recognized that VDD can be responsible for the development of several extraskeletal pathologies, in addition to rickets and osteomalacia, such as multiple sclerosis, autoimmune disorders, the incidence of infections, respiratory diseases, cardiovascular disorders, and various types of cancers [1]. The development of VDD poses a particular risk to individuals with limited intake, insufficient exposure to sunlight, or inadequate intestinal absorption [2]. Vitamin D3 is synthesized in the skin by ultraviolet (UV) radiation. Air pollution, a common problem in developing countries, can also contribute to the development of VDD, as particulate matter absorbs UVB radiation or reflects sunlight [3]. At the same time, the habit of dressing may play a role in developing VDD since the synthesis of vitamin D in the skin is hampered [4]. Decreased 7-dehydrocholesterol (7-DHC) in the epidermis, inadequate consumption of certain foods such as dairy products, and impaired vitamin D metabolism in renal and hepatic insufficiency can also lead to deficiency [5]. On the other hand, certain drugs (e.g., immunosuppressants, glucocorticoids, antiepileptics, antifungals, and antiretrovirals) may cause increased catabolism of vitamin D resulting in VDD [1,5,6].

The term “vitamin D” is a general one that refers to several structurally related secosteroids, including cholecalciferol, ergocalciferol, 25-hydroxyvitamin D (calcidiol), or for 1,25-dihydroxyvitamin D (calcitriol) [7]. For the sake of clarity, in this review, the term vitamin D is used to refer to the animal-derived cholecalciferol, or vitamin D3, and to the plant-derived ergocalciferol, or vitamin D2, except when discussing VDD, which is defined based on serum calcidiol levels. All of them are biologically inactive and need activation through a series of enzymatic transformations. The active forms of both compounds (i.e., 1,25-dihydroxy vitamin D3 and 1,25-dihydroxy vitamin D2) are pleiotropic secosteroid hormones, being involved in regulating the expression of more than 900 genes by binding to the vitamin D receptor (VDR, NR1|1) that acts as a nuclear transcription factor after the formation of a VDR/retinoid-X receptor/cofactor complex.

Vitamin D is necessary for all vertebrates, including humans, to maintain adequate phosphate and calcium levels in the blood, thereby helping to develop normal bone, optimal maintenance of muscle contractions, and cellular functions in different parts of the body [8]. Along with the best-known effects of regulating calcium absorption and promoting bone mineralization, the target genes are involved with a wide variety of biological processes, such as cell proliferation and differentiation, immune response and cytokine regulation [9]. In the last two decades, convincing evidence has been published that indicate the implication of the vitamin D signaling system in several brain functions and disorders. First, animal models demonstrated that it regulates the brain’s development in early life and it contributes to synaptic plasticity, neuroprotection, neural circuitry, and turnover of the dopaminergic system in adults [10]. Second, epidemiological data showed an association between low maternal serum calcidiol levels during pregnancy and disorders of cognitive development in offspring [11]. Third, patients affected by neurodegenerative (e.g., Alzheimer’s and Parkinson’s disease), neuroinflammatory and neuropsychological diseases showed decreased serum calcidiol levels [12]. Based on these observations, a vast number of neuropsychiatric illnesses including dementia and cognitive dysfunction were attempted to be treated by vitamin D supplementation, however, there is no evidence that increased intake enhances VDR activation in the human brain [12].

Therefore, the aims of the current review were to discuss the recent evidence of vitamin D signaling in the brain, including its synthesis through alternative pathways and binding to different receptors expressed by neural and glial cells. Furthermore, a critical evaluation of the knowledge gained from animal models and observational studies reporting associations between VDD and cognitive dysfunction has been performed. Finally, the review also briefly addressed the interventional clinical studies investigating the possible roles of vitamin D deficiency and supplementation in the development of cognitive dysfunction in childhood and adulthood. Thus, the authors attempted to summarize the possible pitfalls of translating molecular and preclinical findings into clinical benefits for patients.

## 2. Molecular Evidence of the Relationship between Vitamin D and Cognitive Function

Vitamin D can exert its effect on neurocognition through a number of mechanisms such as induction of neuroprotection, modulation of oxidative stress, regulation of calcium homeostasis, and inhibition of inflammatory processes [10].

Vitamin D acts through its own receptor, VDR, a nuclear hormone receptor found in the central nervous system. VDR has a DNA-binding domain containing two zinc fingers: one of the regions is responsible for DNA binding, and the other is involved in the dimerization of the molecule. The ligand-binding domain at the C-terminal end of the receptor ensures the specificity and selectivity of the response. There is ample evidence that vitamin D exerts a genomic effect on the brain. VDR, found in both developing and adult brains in rodents, has been shown to be present in the neuroepithelium and midbrain of a 12-day-old embryo (E12) [13]. It is present in neurons, glia cells, especially in the temporal, cingulate, and orbital cortex, thalamus, nucleus accumbens, terminal stretch marks, and amygdala, all of which are essential for the development of cognitive functions [14]. It is also expressed in the CA1, CA2, CA3, and CA4 layers of hippocampal pyramidal cells in both rodent and human brains [15]. The distribution of VDR in humans is strikingly similar to that observed in rodents. Its presence in the hippocampus, cerebral cortex, and limbic system of humans and rodents reinforces the role of vitamin D in regulating learning and memory, however, VDR is also localized in the olfactory, visual, and auditory systems, so it may also play a role in somatosensory functions, which could contribute to better cognitive task performances [14,15].

Furthermore, the diverse distribution of VDR in the brain suggests that vitamin D may be involved in neuronal proliferation and stem-cell differentiation. Indirect evidence for the neurodevelopmental role of vitamin D is represented by the morphological changes that occur in offspring of rats subjected to VDD. Eyles and collaborators created the first dietary developmental VDD model, which was characterized by increased brain cell proliferation [16]. On the other hand, developmental VDD has also been demonstrated to affect the expression of genes regulating apoptosis and the cell cycle in rat embryos [17,18]. In a recent study, neuroprotective effects of cholecalciferol have been shown in young rats [19]. Analyzing the development of the whole brain, they described an increase in the volume of lateral ventricles accompanied by a decrease in the hippocampal volume [19]. This finding has not been confirmed in adult mice nor in rats [20,21], but recently it has been described in patients with mild cognitive impairment [22]. In another study, VDD has also been associated with a 28% increase in lateral ventricles in aged humans [23].

On the molecular level, developmental VDD has been proven to affect brain gene and protein expression in the long term. The altered expression of 74 genes was identified, which were supposed to be involved in various neuronal functions [24]. In a proteomic study, 36 proteins involved in calcium homeostasis, neurotransmission, synaptic plasticity, redox balance, oxidative phosphorylation, etc., were dysregulated in the prefrontal cortex and hippocampus of adult animals [25].

VDD has been linked with the dysregulation of dopamine and serotonin neurotransmission. In developing brains, the VDR appears at E12, exactly when the dopaminergic system starts to develop [26,27,28]. Some evidence has demonstrated that VDD affects components of dopaminergic neuron-maturation factors (e.g., reduction in neurotrophin brain-derived neurotrophic factor, TGF-β1 [29], Nurr1 [30] and metabolizing enzymes (e.g., decrease in the expression of catechol-O-methyltransferase [31] and tyrosine hydroxylase [29]). Moreover, Cui et al. demonstrated that calcitriol increased N-cadherin and tyrosine hydroxylase expression in VDR-expressing neuroblastomas, and importantly, VDD reduced their level in embryonic mesencephalon [26,32]. These findings strongly suggest that calcitriol triggers the differentiation of dopaminergic neurons [13].

Serotonin plays a crucial role in brain development because it is a key modulator of neuronal cell proliferation, migration and brain wiring during fetal and early postnatal life [33]. Serotonin turnover may also be influenced due to the regulation of the expression of tryptophan hydroxylase-2 and leptin genes [34,35]. It was found that after short-term exposure to calcitriol, TPH2 expression increased in cultured serotonergic B14 derived from the rat brain [36]. The same study showed that several human-derived cell lines responded to calcitriol treatment in a similar way [36]. Moreover, Jiang et al. have performed a complex study to provide direct evidence to the above-mentioned links between calcitriol–dopamine and calcitriol–serotonin in the brain [37]. They found that chronic calcitriol administration increased the level of γ-aminobutyric acid (GABA), glutamate, but not that of the dopamine and serotonin in the prefrontal cortex and hippocampus. However, dopamine- and serotonin-turnover were increased, which was demonstrated by the high level of 3,4-dihydroxyphenyl acetic acid (DOPAC) and homovanillic acid (HVA) and 5-hydroxyindole acetic acid (5-HIAA), the metabolites of dopamine and serotonin, respectively. On the other hand, they also showed that high expression of the metabolizing enzymes (i.e., catechol-O-methyltransferase for dopamine and monoamine oxidase A for serotonin) could be responsible for the rapid degradation of the neurotransmitter [37].

All the above-mentioned effects of vitamin D are linked to its binding to VDR. However, recently, membrane receptors activated by calcitriol were also identified. The activation of the 1,25D3 membrane-associated, rapid-response steroid-binding protein (1,25D3-MARRS), which also serves as a protein disulfide isomerase A3 (PDIA3), provides a rapid cellular response. PDIA3 possesses several important functions, one of which is to modulate inflammation, apoptosis, and oxidative stress [38]. In rats, the expression of *Pdia3* mRNA is higher in neurons, astrocytes, and endothelial cells compared to the kidney and liver, so they proposed that Pdia3 is the main vitamin D receptor in the rat brain [39]. Early studies in humans suggested that PDIA3 possessed neuroprotective actions against infections or toxic drugs [40,41]. Moreover, exogenous PDIA3 increased the expression of brain-derived neurotrophic factor (BDNF) and phosphorylated cAMP-response element-binding protein (pCREB), thus enhancing cell proliferation in the hippocampus under normal conditions, but it failed to reduce ischemic alterations in gerbils [42]. All these studies supported the hypothesis that activating 1,25D3-MARRS could be involved in calcitriol’s actions. However, surprisingly, the studies conducted on PDIA3 knockout mice reported an attenuated inflammatory response to traumatic brain injury [43]. So, the role of PDIA3 in the cognitive processes is still uncertain, and based on these new results, further research on PDIA3 could be reasonable. Moreover, PDIA3 expression has been proposed as a biomarker in several types of cancer, and increased expression promotes proinflammatory cytokine release [44].

## 3. Transport and Cellular Uptake of Vitamin D

A fully functional brain signaling system involves the presence of ligands in adequate quantity at the site of action. Vitamin D and its metabolites are lipophilic compounds assumed to penetrate the cell membranes and the blood–brain barrier (BBB) by diffusion. According to the free hormone hypothesis, only the unbound fraction (or the free fraction) of total vitamin D metabolites found in blood or extracellular fluid can diffuse into the intracellular space and couple to its receptors and exert its effects. However, all vitamin D metabolites bind to DBP in a very high proportion (~99%) resulting in picomolar concentrations of free fraction [45]. DBP is synthesized in the liver and circulates in high amounts in the serum, but it can also be found in the interstitial space of various organs. DBP is a low molecular-weight protein, smaller than albumin, but it is essential to build a pool of circulating calcidiol, thus preventing the rapid onset of deficiency. On the other hand, DBP-bound vitamin D metabolites are filtered in the renal glomerulus, but these can be reabsorbed by the proximal tubule through endocytosis by the megalin/cubilin complex [46]. Megalin is expressed along with cubilin mainly in the kidney, brain, and eyes. The complex surely has a role in normal brain development, because the decline of its expression is associated with neurodegenerative processes (e.g., Alzheimer’s disease) [47]. Theoretically, megalin can bind and internalize DBP-bound calcidiol, thus facilitating the access of megalin-expressing cells to calcidiol, but the existence of such a transport mechanism has not been demonstrated yet [45]. Therefore, it can be assumed that the free hormone model is applicable and relevant for calcitriol also.

Another important piece of evidence endorsing vitamin D actions in the brain was the detection of vitamin D metabolites in rodents and the human brain [48,49]. Early autoradiographic studies demonstrated that calcitriol penetrates the BBB and is distributed in the brain [50], but until recently, the quantification of endogenous calcidiol or calcitriol has not been performed from brain tissue. Xue et al. described a correlation between serum and brain total calcidiol concentrations in rats, however, the results should be interpreted with caution, considering that no correction was applied to residual blood from cerebral vessels [48]. It is also noteworthy that DBP is found in cerebrospinal fluid (CSF), so vitamin D metabolites are likely to bind to this. However, the expression of DBP in the CNS is influenced by different pathological conditions (e.g., multiple sclerosis, meningitis) [51], so the free fractions of calcidiol and calcitriol may fluctuate and are very difficult to quantify.

## 4. Assessment of the Vitamin D Status

In the human body, the main metabolic markers used for the assessment of vitamin D status are hydroxylated metabolites, 25-OHD3 and 25-OHD2, due to their long half-lives (2–3 weeks) and stability [52]. In addition, 25-OHD represents the sum of vitamin D intake and dermal synthesis.

The current guidelines suggest that total serum 25-OHD is an appropriate biological marker for assessing vitamin D status, so it can be used to determine vitamin deficiency or sufficient or even excessive vitamin levels [53]. Deficiency may be indicated by total serum levels of <20 ng/mL (<50 nmol/L) for 25-OHD and total serum levels of 25-OHD between 21–29 ng/mL (52.5–72.5 nmol/L) may be considered insufficient without obvious clinical symptoms [1,15]. Vitamin D supplementation is the most widely used strategy to restore vitamin deficiency. The recommended intake is 800 IU to reach 25 ng/mL total serum 25-OHD, 1600 IU vitamin D to 30 ng/mL total serum 25-OHD. The estimated average requirement is 400 IU to reach 20 ng/mL and 800 IU to total serum levels of 30 ng/mL [54].

The serum is the most important medium for the detection of vitamin D metabolites. Calcidiol is the major circulating form of vitamin D in serum. Serum calcidiol levels provide a significant response to changes in vitamin levels during both sunlight and seasonal changes, as well as in vitamin D supplementation [53]. However, the relationship between serum and tissue, especially brain tissue concentrations, is not clarified yet. Furthermore, there is a lack of evidence on the influence of supplementation on brain tissue levels in humans. In rodents, Xue et al. described that the concentration of vitamin D3 metabolites increased in the brain of rats fed with a vitamin D3-rich diet, and they observed that serum and brain levels correlated [48]. However, they quantified only the 25-OHD3 and 24,25-OH2-D3 concentrations, the modification of calcitriol concentration in the brain after vitamin D3 supplementation remains controversial.

One of the challenges with measuring the concentration of calcitriol in brain tissue is its very low level and a lack of quantitation methods that can determine picomolar concentrations. Still, Fu et al. developed a highly sensitive analytical method and detected calcitriol in some regions of the human postmortem brain along with calcifediol [49]. This was the first direct evidence that calcifediol is the most abundant vitamin D3 metabolite not only in the serum but also in the human brain. Cholecalciferol was not detectable, but only one human brain specimen was analyzed, and it was likely from an older subject. So, there is still a need for further investigations on whether cholecalciferol supplementation increases the vitamin D signaling pathway in the brain in order to assess the role of supplementation in certain pathologies. It is important to note that in all available studies, including preclinical and clinical data, VDD was defined based on total serum levels of 25-OHD.

## 5. Synthesis and Catabolism of Calcitriol in the Brain

**Figure 1:**
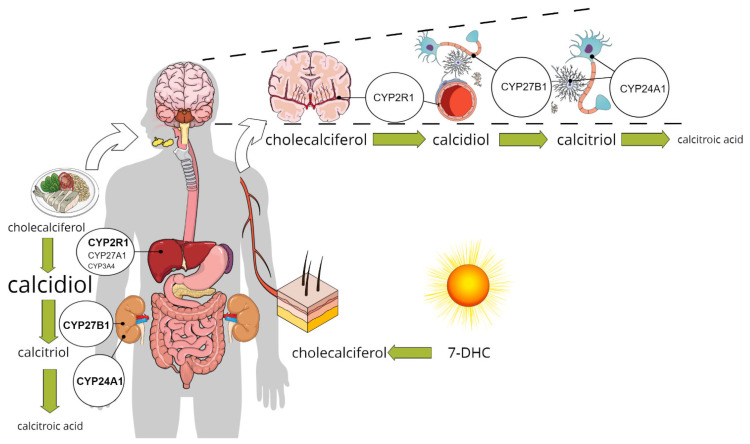
The classical and the alternative pathway for calcitriol synthesis: enzymes involved in calcitriol synthesis are expressed in pericytes, glial cells, and neurons in addition to the liver and kidney, pointing to the possible role for the local production in vitamin D signalling.

The activation of vitamin D receptors (VDR or PDIA3) in different brain regions supposes that calcitriol reaches certain concentrations in the interstitial or intracellular space. As presented above, this can happen if calcitriol passes the BBB and gets access to cells. According to the current knowledge, the synthesis of calcitriol takes place through the classical route that begins in the epithelial layers of the intestine, as cholesterol from food or bile is oxidized to 7-DHC, which is then further activated in the liver and kidney (Figure 1) [55]. Recently, the hypothesis of augmenting the “systemic” calcitriol action by local production has recently been broadened to include the central nervous system [15]. However, the only evidence supporting this is the expression of the enzymes involved in synthesis and catabolism of calcitriol by neurons, glial cells, and microglia. There is no direct evidence for local production or catabolism of calcitriol in different types of brain cells. In this chapter, the expression of the key enzymes will be discussed, highlighting the latest results regarding their expression in the brain.

In the skin, the UVB radiation breaks the bond between C9 and C10 atoms, resulting in an unstable 9,10-secosteroid known as pre-vitamin D3. At body temperature, double bonds of pre-vitamin D3 undergo thermal isomerization, as removal of the bond allows pre-vitamin D3 to spontaneously rotate around the bond between C5 and C6 atoms to form a more thermodynamically stable isomer, vitamin D3 or cholecalciferol [55]. Vitamin D3 is still an inactive precursor that needs to go through a two-step hydroxylation to be converted to calcitriol, a biologically active compound that is able to bind to VDR. Either dietary vitamins (D2 or D3) or cholecalciferol synthesized in the skin binds to a transport protein, the vitamin D binding protein (DBP), and is distributed by the circulation to the liver or other tissues that can convert it further [56]. The so-called vitamin D 25-hydroxylases include six enzymes of the cytochrome P450 enzyme-family (CYP27A1, CYP2R1, CYP2J2/3, CYP3A4, CYP2D25, and CYP2C11), which produce the hydroxylated form at position C25. Their distribution is organ- and organelle-specific: only one of them (CYP27A1) is located in the mitochondria, and the other five are microsomal enzymes [57,58]. In the liver, where the highest amount of 25-hydroxyvitamin D3 (25-OHD3 or calcidiol) is formed in all species, the CYP2R1 and the CYP27A1 are considered the most important [59]. CYP27A1 can form 24-hydroxylated or 26-hydroxylated products also, while the regioselectivity of CYP2R1 is clearly limited to C25 [60]. CYP3A4 might also catalyze the hydroxylation of several forms of vitamin D (i.e., vitamin D3, vitamin D2, 1α(OH)D3, and 1α(OH)D2) in more than one position. It has also been shown to act as a 24-hydroxylase toward 1α(OH)D3, 1α(OH)D2, and vitamin D3, so not only the activation but also the clearance of vitamin D could be influenced by CYP3A4. Drugs that strongly induce CYP3A4 were demonstrated to provoke VDD [61]. Members of the CYP2J and CYP2D families were linked to 25-hydroxylation, but neither the specificity nor the affinity of these enzymes was considered highly important [58].

Thus, it can be concluded that in humans, the CYP2R1 is mainly responsible for calcidiol synthesis, but other 25-hydroxylases also contribute, especially at increased substrate levels [59,62].

In the brain, the expression of CYP2R1 was demonstrated in pericytes, which may play a significant role in regulating the permeability of the BBB for vitamin D metabolites and suggests the existence of a neurovascular vitamin D autocrine/paracrine system [63]. Furthermore, CYP3A4 also shows a cell type- and region-specific expression in the brain which might have new roles beyond drug clearance [64,65].

The next step of the activation of vitamin D3 is catalyzed by 1α-hydroxylase (CYP27B1) at the C1 position of 25-OHD3, resulting in 1,25-dihydroxy-vitamin D3 [58,66]. The transformation of vitamin D2 from plant sources takes place in a similar way. 1α-hydroxylase, or CYP27B1, is a mitochondrial enzyme and a protein of 507 amino acids, with a molecular weight of approximately 55 kDa. Its expression is mainly in the proximal tubule of the kidney and is stimulated by parathyroid hormone (PTH), low calcium and phosphate serum levels, and inhibited by fibroblast growth factor 23 (FGF-23) [58].

It is important to note that the expression of CYP27B1 was demonstrated to take place in the brain as well as the epithelial cells of different organs (breast, prostate, eye), the placenta, bone cells, macrophages, T and B cells and various endocrine glands [67]. However, whether the enzyme CYP27B1 has a functional effect in vivo at sites outside of the kidney and placenta under normal physiological conditions remains questionable.

In a comprehensive study, Landel et al. observed that not only are the receptors of vitamin D (VDR and Pdia3) expressed in different brain cell types, but also the mRNAs of the enzymes involved in vitamin D synthesis and metabolism are highly expressed [39]. They proposed that endothelial cells and neurons can transform cholecalciferol to calcidiol as these cell types highly express *Cyp27a1*. Furthermore, neurons and possibly microglia might be able to activate calcidiol to calcitriol, because these cells have had a considerable expression of *Cyp27b1* (Figure 1) [39]. This was also confirmed in foetal and adult human brains, suggesting local production of calcitriol might be possible [68].

The metabolism of vitamin D also includes inactivating transformations of each intermediate compound. The most important enzyme is the 25-hydroxyvitamin D3 24-hydroxylase, or CYP24A1, responsible for the side-chain hydroxylation of both 25-OHD3 and 1,25-(OH)2D3 [69,70]. Both reactions lead to calcitroic acid, which is excreted in bile, although some evidence suggests that these 24-hydroxylated compounds may have activity [60,71,72]. The reorientation of the C3-beta hydroxyl group to the C3-alpha position also reduces the biological activity of vitamin D metabolites. It is catalyzed by 3-epimerase, which transforms about 4% to 10% of the circulating calcidiol to 3-epi-calcidiol [58].

Another important catabolic process includes the C-23 oxidation pathway, which results in the formation of 1,25-dihydroxyvitamin D-26,23-lactone from the 1,25-(OH)2D substrate and 25-hydroxyvitamin D-26,23-lactone from calcidiol. The 1,25-dihydroxyvitamin D-26,23-lactone formed in this pathway acts as a VDR antagonist [73].

Calcitriol regulates its own biosynthesis by inducing CYP24A1 expression in the kidney [70]. The importance of CYP24A1 in the regulation of vitamin D activity has been demonstrated by several reports showing that CYP24A1 mutation leads to excessive calcitriol activity, resulting in hypercalcemia, hypercalciuria, nephrocalcinosis, and nephrolithiasis. In contrast, enzyme overactivity is associated with various diseases, such as hyperproliferative disorders and chronic kidney disease [74].

Interestingly, *Cyp24a1* expression has also been detected in cortical and hippocampal neurons and microglia isolated from rat brains, but it was undetectable in astrocytes [39]. However, when treated with calcitriol, astrocytes expressed a higher mRNA level of *Cyp24a1,* suggesting that a calcitriol-induced regulatory feedback mechanism is dependent on VDR signaling, with astrocytes expressing the VDR at the highest levels [39]. This was also shown at a protein-expression level, with VDR and CYP24A1 being upregulated by calcitriol in primary human astrocytes [75].

To summarize, all the necessary elements of vitamin D signaling have been found in the brain, so an autocrine/paracrine functionality could theoretically be assured by precursors, activating or inactivating enzymes of calcitriol, but further studies are warranted to elucidate whether CYP2R1, CYP27a1, and Cyp24a1 enzymes are expressed at protein levels also, or if post-translational events occur which could also regulate vitamin D synthesis and catabolism in the brain.

## 6. Influence of Vitamin D3 Deficiency/Supplementation on Cognitive Impairment in Animal Models

The behavioral effects of manipulating vitamin D signaling on brain development have been studied extensively by using genetic knockout (VDR−/− or CYP27B1−/−) models and models of dietary deficiency. Dietary deficiency models might seem to have more construct validity but are time-consuming, labor-intensive, and can be limited in result interpretation.

Eyles et al. proposed a simple dietary model of developmental VDD: female rats were housed in UV-free conditions and supplied with a vitamin D deficient diet for six weeks [18]. This procedure consistently produced a decrease in serum calcidiol level. Moreover, calcitriol levels were also reduced during gestation depending on the timing of normal diet reintroduction [18,76,77,78]. In most of the studies, they reported no differences in calcium and phosphate serum levels, but in one of the experiments, PTH levels were increased in the VDD group [76]. Moreover, in some studies, no data were provided because sampling-induced stress could have been a potential confounding factor [78,79,80]. However, it should be noted that calcitriol deficiency can only be achieved when serum calcidiol levels fall below 10 nM, because animals with normal renal function can synthesize adequate levels of calcitriol [81]. In this case, secondary hyperparathyroidism can only be avoided by increasing the dietary calcium content [82,83].

The behavior of rats and mice subjected to dietary developmental VDD has been observed in various experimental conditions. In rats, developmental VDD induced disrupted latent inhibition, which reflects the attentional processing of animals [78] but normal prepulse inhibition and working memory. However, these animals showed hyperlocomotion and increased exploratory activity, which might enhance some types of cognitive functions or could be a confounding factor in several behavioral tests used to assess cognitive function [78,84]. Indeed, electrophysiological studies demonstrated an enhanced long-term potentiation in the hippocampus and better retention performance in the Y-maze test in prenatal VDD animals, both results supporting an improved memory formation in rats [82,85]. Conversely, mice exhibited learning deficits, although they showed the same increase in exploration and hyperlocomotion as rats [77,80,86]. This means that developmental VDD might influence the various components of cognition differently. Using complex behavioral paradigms, such as the five-choice serial reaction time task (5C-SRT) and the five-choice continuous performance test (5C-CPT), which can reflect the sustained attention and vigilance in rodents, VDD rats had normal performance to target stimuli and maintained high levels of accuracy but had increased probability of false-alarm responding to nonsignal stimuli, and they showed poorer vigilance and a lower responsivity index [87]. In mice, VDD mice showed similar results to controls in the primary measures of performance but tended to make more premature responses than control mice [80]. These subtle alterations observed in rats and mice may represent a symptom of a compulsive or impulsive behavior rather than cognitive dysfunction. Recently, Overeem et al. described altered recognition memory associated with developmental VDD [79], however, the results obtained in the novel object recognition test need further confirmation as this test has a wide range of manipulations, and it also has its limitations [88]. Overall, the cognitive impairments reported in the developmental VDD model may be associated with psychiatric disorders such as schizophrenia, autism, or attention deficit hyperactivity disorder, particularly because some of them can be treated with different antipsychotics [84,85,87].

Besides developmental VDD models, several genetic mice models were obtained by VDR gene ablation or mutation in mice [89,90,91]. These animals showed increased levels of PTH and calcitriol along with hypocalcemia, hypophosphatemia, and consequential skeletal disorders [92]. Therefore, to study the abnormalities induced by VDR ablation only and not the consequences of altered mineral homeostasis, a calcium- and phosphate-rich diet, a so called “rescue diet”, should be delivered to VDR knockout animals [90,92,93,94]. Even so, a great variation in PTH values (from normal to excessive PTH) has been observed along with VDR-independent compensatory mechanisms [95,96]. On the other hand, increased dietary calcium load induces FGF23 synthesis [97], which was shown to affect spatial learning and memory [98]. Thus, the contradictory results reported so far between dietary and genetic VDD models may be due to several confounding factors, one of which is the FGF23 [99].

Taking into account the above-mentioned endocrinological modifications induced by VDD knockout, the altered behavioral responses are difficult to understand. Burne et al. and Kalueff et al. studied thoroughly the behavioral anomalies caused by the lack of functional VDR in mice and reported an array of neuropsychiatric deficits (e.g., anxiety, neophobia, altered maternal behavior, impaired motor coordination), along with muscle weakness, impaired energy metabolism, and altered cardiac functions [100,101,102,103,104,105,106]. As all these alterations affect the performance of mice in a behavioral test, the VDR knockout model is not suitable to study the impact of calcitriol on cognition.

Various preclinical models were proposed to study the influence of transient VDD and vitamin D3 supplementation on adults. Hypocalcemia and secondary hyperparathyroidism consecutive to VDD induction have been addressed in different ways by the research groups. Burne’s group applied a vitamin D-free diet for six weeks in rats and ten weeks in different strains of mice and demonstrated an up to a 10-fold decrease in the serum calcidiol levels, below 10 nM in each experiment, associated with normocalcemia [107]. However, they did not modify the calcium or phosphorus intake of the animals and did not monitor the serum PTH levels. Nevertheless, they found that this protocol is not sufficient to induce cognitive alterations in the young adult rat, observing a slight effect on vigilance only [107]. On the other hand, in ten-week-old mice, spontaneous hyperlocomotion was described similarly to developmental VDD models, but there was no influence of the diet on cognitive functions. Neither the old mice nor the middle-aged rats showed cognitive impairment, even though they had been deprived of vitamin D for more extended periods of time (6–12 months) [108,109]. One specific learning task did appear to be influenced by VDD in mice, namely the hippocampal-dependent spatial learning. VDD mice exhibited impairments in the active place-avoidance test, which were not linked to motor coordination problems or muscle weakness. It is important to note that connectivity deficits were also observed in the hippocampus [59]. Latimer et al. reported that in middle-aged rats subjected to 6-months of vitamin D manipulation through diet, serum calcidiol levels correlated with the performance of the animals in the Morris water maze task, and supraphysiological doses may be protective against mild cognitive impairment [110]. Liang et al. performed a similarly designed study in mice, including a group with a supraphysiological dose of vitamin D. In their study, mice were fed life-long with a specific diet containing a higher level of calcium before they were behaviorally assessed at 6-weeks and 17-weeks of age [111]. They concluded that altered vitamin D intake had a significant impact on long-term memory and promoted memory loss (not only VDD but also the overdose were harmful) [111].

A different approach to study the effect of vitamin D supplementation was also proposed. Stavenuiter et al. demonstrated that vitamin D depletion could be obtained rapidly if several doses of paricalcitol were administered to rats [89]. Paricalcitol is a potent CYP24A1 inductor that provokes rapid catabolism of calcidiol and calcitriol. Three weeks after induction, the serum calcidiol and calcitriol levels were below the detection limits, but no elevation in PTH serum levels has been detected [89]. Various aspects of vitamin D signaling has been studied using this model (e.g., bone and mineral metabolism, thyroid function changes, hypertension, oxidative stress, and inflammation), only the CNS characteristics remained to be described.

**Figure 2:**
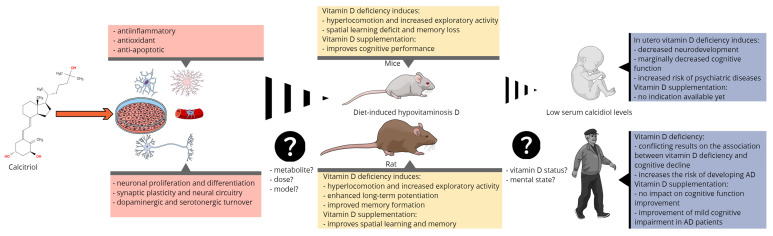
Summary of the main molecular, preclinical, and clinical findings linking vitamin D to cognitive function and the most important issues of translational research.

In summary, animal models of VDD have shown that low total serum calcidiol induces behavioral changes, suggesting that calcitriol is involved in a number of important neuronal and glial processes. Interestingly, VDD did not cause cognitive impairment in rodents as would be expected, but vitamin D supplementation improved some of their cognitive processes (Figure 2). Possible explanations for the controversial finding may be related to study design (i.e., animal model, diet, dosing, paraclinical tests used to confirm VDD), but some unknown aspects of vitamin D metabolism in the brain may also play a role.

In several studies, adult VDD models have been combined with other uniquely human psychiatric conditions such as schizophrenia or depression to study the influence of VDD as an associated risk factor. The co-occurrence of VDD with other psychiatric disorders showed that the increased corticosterone response to stressful events might be aggravated, as reflected by the increased avoidance times observed in mice that underwent a social interaction test [112]. Many findings sustain that VDD is a significant risk factor associated with neuropsychiatric diseases, but this topic is out of the scope of this review. These issues were reviewed elsewhere [113,114].

## 7. Influence of Vitamin D3 Deficiency/Supplementation on Cognitive Impairment in Humans

### 7.1. Risk Associations between Maternal Vitamin D Status and Neurocognitive Development

In parallel with the discovery of the presence of VDR in the central nervous system and vitamin D’s potential impact on neurodevelopmental processes in animal models, epidemiological studies have emerged to establish whether there is an association between prenatal vitamin D exposure and brain development outcomes in humans.

The effects of prenatal VDD on several aspects of cognitive functions have been extensively studied in children, but the results obtained are controversial. The most important sources of variations are the duration of the follow-up, instruments and definitions of neurodevelopmental outcomes, the timing and method of vitamin D status assessment, and the lack of data regarding the infant and child vitamin D status. In this review, we proposed to subclassify the existing evidence based on the follow-up period (i.e., whether the cognitive functions have been assessed in infancy or in early childhood) in order to exclude the bias induced by comparing data obtained at all ages. Thus, we focused on large-scale prospective pregnancy cohort studies which assessed offspring for neuropsychological development in the first two years of age (ranging from 6 months to 24 months).

There are five high-quality studies that were conducted on infants using similar tests and instruments in the assessment of offspring’s neurocognitive status. These studies reported data obtained by psychologists or certified examiners [115,116,117,118,119] (Table 1). In infants examined at 11–23 months of age, a significant association between maternal calcidiol levels and mental and psychomotor development was observed in a Spanish population-based cohort study, and infants of mothers with calcidiol concentrations in pregnancy >30 ng/mL showing higher mental score [115].

Hanieh et al. reported a high prevalence of VDD among pregnant Vietnamese women, and low maternal 25-hydroxyvitamin D levels measured during late pregnancy resulted in reduced language developmental outcomes at six months of age [116]. Similarly, a large-scale cohort study from the USA involving a racially diverse population revealed that gestational 25(OH)D status (measured in the 2nd trimester) is positively associated with receptive-language scaled scores at the age of 2 years (*p* < 0.017), but not with cognitive- or expressive-language scaled scores in multivariate analyses [118].

However, Keim et al. conducted the largest case–control study on this topic and measured 25(OH)D circulating levels both in mothers and newborns. They observed no consistent associations between maternal cord blood vitamin D concentration and achievements on cognitive tests at different time points (8 months, 4 and 7 years). These results were tested upon adjusted models including a series of potential confounders, however, one major limitation, that data originates from the 1960s, should be considered [117].

On the other hand, a Chinese study conducted on toddlers aged 16–18 months found interesting results when testing the relationship between neonatal vitamin D status and neurodevelopmental outcomes [119]. In this study, newborn cord blood samples were collected during delivery, which is known to correlate as 75–90% of maternal concentrations. According to cord blood 25(OH)D concentration, the cohort was divided into quintiles. Unexpectedly a nonlinear trend in the mental and psychomotor development indexes was observed throughout the quintiles, with the highest scores measured in the 3rd and 4th quintiles. Furthermore, toddlers in the lowest and highest quintiles presented a significant deficit in neurodevelopment (*p* < 0.001). Further studies are needed to confirm the optimal levels of neonatal vitamin D with respect to later neurocognitive development outcomes. Based on the results of this study, it might range from 30 to 50 nmol/L (the third and fourth quintile) [119].

Findings are similar in studies conducted in later childhood. In an Australian birth cohort study, maternal VDD was associated with neurocognitive difficulties in 10-year-old children [120], but in a similar study conducted in Denmark, the scholastic achievement for offspring of mothers with VDD was not statistically different. However, in the same study, a significant association between maternal vitamin D status and offspring depression was observed [121]. Taken together, the diversity of the methodology of neurodevelopment assessment and the multifactorial origins of cognitive impairments makes it difficult to find a link between VDD and cognitive performance in children. Some studies handled a great number of covariates, which also impedes the direct comparison of the results and their interpretation (Table 1). 

In a recently published meta-analysis and systematic review, Garcia-Serna and Morales, 2020, presented a thorough analysis of all possible consequences of VDD, including behavioral difficulties, ADHD, and autism spectrum disorder [11]. They found only a borderline positive association between 25(OH)D levels and cognitive development, but a strong inverse association between 25(OH)D levels and risk of ADHD and autism in offspring. So, they concluded that higher prenatal 25(OH)D circulating concentrations might reduce the risk of ADHD and autism-related traits [11]. This hypothesis has already been sustained by some data from maternal–fetal studies [122].

In conclusion, we could state that the currently available evidence is limited regarding the establishment of optimal levels of circulating vitamin D in maternal blood and achieving the best neuropsychological outcomes in infants. There seems to be a trend towards a beneficial effect of vitamin D in early pregnancy, known to be the most critical period in human neurodevelopment and a protective effect against the development of ADHD and autism.

### 7.2. Risk Associations between Vitamin D Status and Cognitive Decline

The neuroprotective, anti-inflammatory and antioxidant properties of vitamin D have been widely studied in preclinical studies, which then led to the hypothesis that vitamin D could serve as a potentially modifiable risk factor for cognitive decline and neurodegenerative diseases. Clinical studies are, however, contradictory.

To date, the association between VDD and prevalent dementia and neuroimaging abnormalities is based mainly on observational data (cross-sectional and case–control studies) with major bias and limitations. These studies are designed based on very different criteria and short follow-up periods, which may lead to incorrect conclusions given that most types of dementia have a slow progression over time. Potential confounders and the various factors reducing randomized controlled trials’ (RCT) specificity has been discussed thoroughly elsewhere [123]. Furthermore, the lack of a standardized method to measure serum vitamin D concentrations due to analytical challenges [124] and the multitude of tests used to assess the cognitive status make it difficult to interpret the results of different studies (Figure 2).

We aimed to review clinical studies with longer follow-up periods and significant or representative sample sizes (>500 subjects), mainly large national cohorts and community-based samples (those which used similar diagnostic criteria, assessments to identify impaired cognition and consistent categorization of serum vitamin D levels). However, the scientific literature is lacking regarding standardized prospective studies and RCTs in the human population.

#### 7.2.1. Observational Studies

Two large-scale prospective studies from European countries suggested that low vitamin D levels are a predisposing factor for cognitive decline [125,126]. Both studies were testing a large community-based elderly population aged >55 years in the Rotterdam study and >65 years in the French one, without any evidence of dementia at the time of recruitment. Both study groups were monitored for serum vitamin D levels and cognitive status, VDD being defined as serum levels <25 nmol/L. The strength of the Rotterdam study is the cohort size (n = 6220 subjects) and long follow-up period (13.3 years), as well as the use of appropriate regression models and adjusted analyses to exclude confounding factors such as age, sex, ethnicity, season, serum calcium and kidney function. Licher et al. revealed a trend toward increased risk of developing dementia in subjects with vitamin D < 25 nmol/L (defined as the deficiency) when compared to those with ≥50 nmol/L (sufficiency) without reaching statistical significance [125]. However, lower vitamin D concentrations were associated with a higher risk of dementia (adjusted HR, per SD decrease 1.11, 95% CI 1.02; 1.20) and Alzheimer’s disease (adjusted HR: 1.13, 95% CI 1.03; 1.24) in the longitudinal analyses [125].

Another large population-based study from Denmark (n = 10,186 subjects, follow-up 30 years) reported a positive association of reduced plasma 25(OH)D levels with increased risk of developing Alzheimer’s disease and vascular dementia (HR for the combined endpoint 1.27 (95% CI, 1.01–1.60)) [127]. Similarly, Littlejohns et al. analyzed risk associations from the US population-based Cardiovascular Health Study data [128]. A total of 1658 elderly ambulatory adults free from dementia, cardiovascular disease, and stroke were included in the study, which revealed that the risk of developing all-cause dementia is higher in participants who were either 25(OH)D-deficient or severely deficient (*p* = 0.002) [128].

On the contrary, there are studies reporting no association between vitamin D status and risk of developing any type of dementia (Table 2). Karakis et al. analyzed cohorts from the US Framingham Heart Study and found no associations between vitamin D levels and incidence of Alzheimer’s disease [129]. This study included 1663 subjects aged >60 years, nondemented, and almost all Caucasian who underwent a complex cognitive status assessment using neuropsychological testing (Trail Making A and B, Logical Memory, Visual Reproductions delayed, Similarities, and the Hooper Visual Organization Test) alongside MMSE and MRI neuroimaging markers of subclinical brain aging were measured. It is worth noting that the serum 25(OH)D concentration cutoff used to determine VDD was <10 ng/mL [129].

Olsson et al. reported similar findings from a Swedish, prospective community-based study (Uppsala Longitudinal Study of Adult men; n = 1182), however, the use of a VDD cutoff of 50 nmol/L should be taken into consideration [130]. A recently published 10-year cohort study from the Canadian Study of Health and Aging followed 661 nondemented older adults for more than five years, but no significant association between 25(OH)D and cognitive decline, dementia or AD was found [131].

**Table 2:** Prospective studies on association between vitamin D status and cognitive decline.

Study	Study Design and Follow-Up	Population	Methods	Results	Conclusion
D Lee et al., 2020, Korea [132]	Prospective Korean frailty and aging cohort study Duration of follow-up not reported	N = 2990 subjects (1415 men and 1575 women) 2 years of baseline data	Assessment of cognitive status-tests not reported Serum 25(OH)D concentration (CMIA method) VDD cutoff: 10 nmol/L	119 (4.0%) VDD; 2253 (75.3%) insufficient Only 618 (20.7%) participants were sufficient for 25(OH)D	Better performance in cognitive tests in sufficient > insufficient > deficient groups (*p* < 0.05) No direct correlation between vitamin D levels and cognition
C Duchaine et al., 2020, Canada [131]	Prospective Canadian Study of Health and Aging 10-year cohort study Follow-up: 5.4 years	N = 661 subjects aged >65 years without dementia at baseline	Assessment of cognitive status (3MS) Serum 25(OH)D concentration (CLIA method) VDD cutoff < 25 nmol/L	141 subjects developed dementia of which 100 were AD.	No significant association between 25(OH)D and cognitive decline, dementia or AD.
C Feart, 2017, France [126]	Prospective Three-City Bordeaux cohort Follow-up: 12 years	N = 916 subjects aged >65 years, nondemented at baseline	Cognitive status not reported Serum 25(OH)D concentration (CMIA method) VDD cutoff < 25 nmol/L	VDD was associated with a nearly three-fold increased risk of AD (HR = 2.85, 95% CI 1.37–5.97).	Association between lower vitamin D concentrations and increased risk of AD.
Licher, 2017, Netherlands [125]	Prospective Follow-up 13.3 years	N = 6220 subjects aged >55 years, 6087 nondemented at baseline	Cognitive status- MMSE, GMS Serum 25(OH)D concentration (ECLIA method) VDD cutoff < 25 nmol/L	795 participants developed dementia, of whom 641 had AD. Lower vitamin D concentrations were associated with higher risk of dementia (adjusted HR, per SD decrease 1.11, 95% CI 1.02;1.20) and AD (adjusted HR: 1.13, 95% CI 1.03;1.24).	Lower vitamin D concentrations increase the risk of developing AD
Olsson, 2017, Sweden [130]	Prospective Community based (Uppsala Longitudinal Study of Adult men)	N = 1182 subjects, men only Mean age 71 years Follow-up12 years (mean)	Cognitive status-MMSE Serum 25(OH)D concentration (HPLC-MS) VDD cutoff 50 nmol/L	116 cases of AD, 64 cases of vascular dementia, and 250 cases of all-cause dementia identified. Eighty of 488 men who participated in the MMSE at follow-up were classified as cognitively impaired (16.4%).	No association between baseline vitamin D status and long-term risk of dementia
Karakis, 2016, USA [129]	Prospective Framingham Heart Study cohorts Follow-up 9 years	N = 1663 subjects aged >60 years, nondemented, almost all Caucasian	Cognitive status, MMSE Complex neuropsychological testing MRI markers of subclinical brain aging were measured Serum 25(OH)D concentration (RIA method) VDD cutoff < 10 ng/mL	Mean 25(OH)D concentrations were 25.1 ± 11.4 ng/mL for the dementia cohort and 19.8 ± 7.4 ng/mL for the cognitive/MRI outcome cohort. 96 (6%) participants in the dementia cohort, and 104 (8%) in the cognitive outcomes cohort were vitamin D deficient.	No associations between vitamin D levels and incidence of AD.
Afzal, 2014, Denmark [127]	Prospective Follow-up 30 years	N = 10,186 subjects Danish general population	Cognitive status- tests not reported Serum 25(OH)D concentration (ECLIA method) VDD cutoff < 25 nmol/L	Positive association of reduced plasma 25(OH)D with increased risk of the combined endpoint of AD and vascular dementia (HR for the combined endpoint 1.27 (95% CI, 1.01–1.60)	Lower vitamin D concentrations increase the risk of developing AD.
Littlejohns, 2014, USA [128]	Prospective Cardiovascular Health Study, a large, population-based study Follow-up 5.6 years	N = 1658 subjects Both men and women, both black and white	Cognitive status-tests not reported Serum 25(OH)D concentration (LC-MS) VDD cutoff 50 nmol/L	The risk of developing all-cause dementia higher in participants who were either 25(OH)D deficient or severely deficient. (*p* = 0.002)	Vitamin D deficiency increases the risk of developing AD
Y Slinin et al., 2010 [133]	Osteoporotic Fractures in Men Study (MrOS), USA	N = 1604 men aged >65 years Follow-up for average 4.6 years	Cognitive status (3MS + Trail Making Test) Vitamin D levels reported in quartiles-4 groups (LC-MS method) (<19.9 ng/mL; <20–25; 25–30; >30)	Lower 25(OH)D level (<19.9 ng/mL) seemed to be associated with greater odds of baseline cognitive impairment	No independent association between vitamin D level and cognitive performance (after adjustments for covariates)

#### 7.2.2. Interventional Studies

To date, there are only a few RCTs available in the scientific literature testing the potential beneficial effects of vitamin D supplements in the prevention of cognitive decline (Table 3). Two recently published RCTs from China used identical intervention plans, i.e., subjects assigned to two arms either receiving 800 IU/day of vitamin D or starch granules as placebo [134,135]. Both studies had a short follow-up period of 12 months only and a relatively small sample size [134,135]. Jia et al. reported significant improvements in plasma Aβ-related biomarker (*p* < 0.001) levels and cognitive tests (*p* < 0.05) in patients with Alzheimer’s disease when comparing the intervention group over the control group [134]. Yang et al. found that vitamin D supplementation for 12 months improved cognitive function through reducing oxidative stress, expressed as increased telomere length in older adults with mild cognitive impairment. However, the study was underpowered due to the sample size; only 183 subjects were recruited and randomized to an intervention and placebo group [135].

The largest randomized, placebo-controlled trial of vitamin D plus calcium therapy, part of the Women’s Health Initiative Randomized Trial from the US, recruited 4143 women aged 65 and older without probable dementia at baseline and followed-up for 7.8 years. Results indicated a similar incidence of cognitive decline in the treatment group compared to placebo [136].

Bischoff-Ferrari et al. conducted a multicenter study with 1900 adults aged 70 years or above within the Do-Health RTC, testing the impact of vitamin D supplementation, omega-3s, and a strength-training exercise program, alone or in combination in a 3-year follow-up. The authors concluded that vitamin D has no impact on cognitive function improvement [137].

In conclusion, it could be stated that vitamin D supplementation failed to prevent cognitive decline in the healthy elderly population and evidence is scant that vitamin D may improve cognition in older people with Alzheimer’s disease. Currently (as per clinicaltrials.gov accessed on 30 June 2021), two active and ongoing RCTs are testing the efficacy of vitamin D supplementation in community-based elderly cohorts, however, these studies are phase II trials aiming to design a potential phase III trial focusing on elderly groups at risk of dementia (NCT03613116 and NCT03096314). 

**Table 3:** Interventional studies on association between vitamin D replacement and cognitive decline.

Study Author, Year	Study Design	Population	Intervention	Results	Conclusion
Rossom et al., 2012, USA [136]	RCT Double blind, placebo controlled WHI Calcium and Vitamin D Trial and the WHI Memory Study.	N = 4143 women aged 65 and older without probable dementia at baseline Follow-up 7.8 years	Group 1: 1000 mg of calcium carbonate combined with 400 IU of vitamin D (3) (treatment) Group 2: placebo.	39 participants in the treatment group and 37 in the placebo group developed incident dementia. Likewise, 98 treatment participants and 108 placebo participants developed incident MCI	No association between treatment assignment and incident cognitive impairment.
Jia et al., 2019, China [134]	RCT Effects of vitamin D supplementation on cognitive function and blood Aβ-related biomarkers in older adults with Alzheimer’s disease	N = 210 AD patients	12-month treatment in two arms 800 IU/day of vitamin D vs. starch granules as placebo	Significant improvements in plasma Aβ biomarker (*p* < 0.001) levels and cognitive tests (*p* < 0.05) in the intervention group over the control group	Daily oral vitamin D supplementation for 12 months may improve cognitive function and decrease Aβ-related biomarkers in elderly patients with AD
DO-HEALTH Trial Bischoff- Ferrari et al., 2020, Multicenter study [137]	RCT to test whether vitamin D, omega-3s, and a strength-training exercise program, alone or in combination, improved six health outcomes among older adults.	N = 2157 adults recruited aged >70 years No major health events in the 5 years prior to enrolment + sufficient mobility and good cognitive status N = 1900 completed the study	3 years of intervention Eight groups: 2000 IU/d of vitamin D3 1 g/d of omega-3s + a strength-training exercise program (n = 264); vitamin D3 + omega-3s (n = 265); vitamin D3 + exercise (n = 275); vitamin D3 alone (n = 272); omega-3s + exercise (n = 275); omega-3s alone (n = 269); exercise alone (n = 267); or placebo (n = 270).		No significant difference in improvement in systolic or diastolic BP, physical performance, infection rates, or cognitive function after treatment with vitamin D3, omega-3s, or a strength-training exercise
Yang et al., 2020, China [135]	RCT 12 months follow-up	N = 183 subjects Tests of cognitive function (FSIQ, information, digit span, vocabulary, block design, and picture arrangement) were evaluated at baseline, 6 months, and 12 months	Two arms intervention group (vitamin D 800 IU/day, n = 93) placebo group (the matching starch granules, n = 90)	Improvements in the cognitive tests in the vitamin D group over the placebo group (*p* < 0.001)	Vitamin D supplementation for 12 months appears to improve cognitive function through reducing oxidative stress
Zajac et al., 2020, Australia [138]	RCT testing the effects of vitamin D on cognition and mood	N = 436 healthy older male (49%) and female volunteers aged ≥60 years follow-up: 6 months	Four arms- vitamin D3 (D3) enhanced vitamin D2 in a mushroom matrix (D2M), standard mushroom (SM) and placebo (PL)	Levels of total 25-OH-D and 25-OH-D3 were maintained in the D3 arm but decreased significantly (*p* < 0.05) in the remaining arms (D2M, SM and PL).	No significant effects of treatment on any of the measures of cognitive function or mood

RCT—randomized controlled trial; 3 MS—Modified Mini-Mental State; MMSE—Mini-Mental State Examination; CLIA—Chemiluminescence immunoassay; CMIA—Chemiluminescent Microparticle immunoassay; ECLIA—Electrochemiluminescent immunoassay; HPLC—High-performance liquid chromatography–mass spectrometry; LC-MS—Liquid chromatography–tandem mass spectrometry; FSIQ—the full-scale intelligence quotient; WHI—Women’s Health Initiative Randomized Trial.

## 8. Conclusions

The evidence reviewed above demonstrates that vitamin D is important for normal brain development and function in rodents and humans, as deficiency can affect cognition. However, vitamin D supplementation produced conflicting results on cognitive performance, suggesting that some crucial elements of vitamin D signaling in the brain are still uncovered. First, it is not yet elucidated whether there is a critical threshold for total serum calcidiol levels at which brain function alters. Second, no evidence has yet been published that cholecalciferol supplementation increases brain calcitriol levels, so the relevance of such intervention for the prevention or treatment of high-burden neuropsychiatric conditions remains to be established. Third, new hypotheses suggesting a role for PDIA3 as a membrane receptor of calcitriol, as well as local synthesis and catabolism in the brain, further complicate the understanding of vitamin D signaling. Moreover, the activity of lesser-known metabolites (e.g., C-3 epimers, calcitroic acid) should be further investigated as they may contribute to the effects of calcitriol actions in the brain.

On the other hand, there may be methodological reasons for the controversial results reported in clinical studies. There is a clear need to standardize not only the bioanalytical determination methods used to evaluate vitamin D status but also the neuropsychiatric assessment of study subjects in order to find a relationship between VDD and cognitive impairments. Perhaps both the interindividual and interstudy variability can be reduced by finding new ways to assess vitamin D status, such as using free hormone measurements instead of total hormone concentrations or combining total serum calcidiol with serum DBP levels.

Finally, until further data become available by conducting properly designed, high-quality RCTs to explore the therapeutic significance of vitamin D in brain-related disorders, it should be mentioned that there is no recommendation to use vitamin D supplementation in pregnancy to promote normal cognitive development. Older adults should be screened for VDD because calcitriol has multiple beneficial roles in neurodegenerative diseases (i.e., anti-inflammatory, immune-modulatory, and antioxidant actions). However, many observational studies found a nonlinear association between total serum calcidiol concentration and different outcome measures, so there would be no benefit to achieve supraphysiological levels.

## Figures and Tables

**Table 1:** Prospective studies on associations between prenatal vitamin D status and neurodevelopment.

Author, Year [Ref]	Study Design/Population	Vitamin D Levels	Neurodevelopmental Assessment/Age	Covariates	Conclusion
Morales et al., 2012 [115] Spain	Prospective Cohort (2003–2008) N = 1820 mother–infant pairs	Maternal (prenatal at week 13) Cutoffs Per 10 ng/mL <20 ng/mL 20–30 ng/mL >30 ng/mL MS-HPLC method	MDI and PDI assessed by psychologists using the BSID scale (at 14 months)	Area of study, child’s gender, birth weight maternal country of origin, age, social class, education level, parity, prepregnancy BMI, and smoking and alcohol consumption in pregnancy	Positive linear relationship between maternal 25(OH)D(3) concentrations in early pregnancy and mental and psychomotor scores in the offspring.
Hanieh et al., 2014 [116] Vietnam	Prospective Cohort (2010–2012) N = 960	Maternal (prenatal at week 32) Per 10 ng/mL <15 ng/mL 15–30 ng/mL 30 ng/ml	MDI and PDI assessed by psychologists using the BSID scale Language score and socioemotional score (at 6 months)	Maternal age, education, month of sampling of vitamin D, micronutrient intervention, maternal BMI, gravidity, postpartum depression and clustering at commune level	Low maternal 25-hydroxyvitamin D levels during late pregnancy are associated with reduced language developmental outcomes at 6 months of age
Keim et al., 2014 [117] USA	Case-control (1959–1973) N = 3308 N = 1867	Maternal (<26 weeks) and cord blood: Per 2 ng/mL <10 ng/mL 10–20 ng/mL 20–30 ng/mL >30 ng/mL or LC-MS method	MDI and PDI assessed by psychologist using the BSID scale; Stanford–Binet Intelligence Scale; WISC: Global IQ • Rating system behavior: Internalizing & Externalizing behaviors (at 8 months; 4 and 7 years)	Maternal education, age, parity, race, maternal BMI, marital status, smoking, gestational age and month of blood draw, and study site	Very little indication that maternal or cord blood 25(OH)D are associated with cognitive development, achievement, and behavior between 8 months and 7 years of age.
Tylavsky et al., 2015 [118] USA	Prospective Cohort (2006–2011) N = 1020 mother– child dyads	Maternal (2nd trimester) Per 10 ng/mL <20 ng/mL >30 ng/mL EIA method	MDI assessed by the Bayley Scales of Infant and Toddler Development (Bayley-III) Receptive-Language Scaled Score Expressive-Language Scaled Score (at 2 years)	Tobacco use during pregnancy, total number of pregnancies, maternal IQ, gestational age at birth, and race	Higher in utero 25(OH)D exposures during the second trimester were positively associated with receptive language skills in infants at 2 years.
Zhu et al., 2015 [119] China	Cohort (2008) N = 363 mother–infant pairs	Newborn cord blood: Quintile 1 (<8.3 ng/mL) Quintile 4 (15.9–20.4 ng/mL) RIA method	MDI and PDI assessed by certified examiners using the BSID scale (at 16–18 months)	Maternal sociodemographic characteristics, health status, prepregnancy lifestyle, birth outcomes, breastfeeding, and maternal depressive symptoms at 3 months postpartum	Nonlinear (inverted-U-shaped) relation between neonatal vitamin D status and neurocognitive development in toddlers

MDI—Mental development index; PDI—Psychomotor development index; BMI—Body mass index; BSID—Bayley Scales of Infant Development; WISC—Wechsler Intelligence Scale for Children; RIA—Radioimmunoassay; EIA—enzyme immunoassay; LC-MS—liquid-chromatography–tandem mass spectrometry.
